# The Clean Milk Demonstration

**Published:** 1921-02-19

**Authors:** 


### THE CLEAN MILK DEMONSTRATION.

Stand No. 103 at the present Exhibition is one that should on no account be missed. It forms a first-class object-lesson in the hygienic production and distribution of milk. The appeal is not to the general public alone, but to every person who in any way deals with milk up to the time of its actual use as an article of diet, and, from the farmer to the children's nurse, all will find points to hold their attention. Organised by the Ministry of Health, in conjunction with Mr. Wilfred Buckley, the leading spirit of the National Clean Milk Society, the exhibit sets forth all the excellent objects of a great campaign.

First, to take the improved conditions possible at the farm, one is shown in practical form the essentials for feeding the animals so that the best quality milk is obtained. Next, both the new and the old, unsatisfactory cow-barns are shown in a series of perfect models. Actual apparatus for removing hair from the cows, rubber gloves to be worn during milking, and a host of details which make for cleanliness are illustrated, together with improved forms of milk-churns which reduce dirt and bacterial contamination to a minimum. If any one doubts the enormous extent of this possible contamination he has only to examine the series of bacterial cultures on the stand, all obtained from typical milk samples, and including disease-producers of the worst kind.

Then, passing from the farm section, one comes to a display of good and bad methods of bottling and distribution. The modern cardboard-capped bottle is eminently satisfactory, but this fact must not be allowed to obscure the possibility of gross milk contamination in the home after the bottle is opened. A remarkably explicit series of photographs shows what *can* happen, and after this inspection the visitor is not long in appreciating the value of ice-chests, new storage methods, and the host of simple home contrivances which are next placed before him. The main object of the exhibit is to arouse public interest to the dangers of impure milk; but not this alone. Cleanliness can be obtained by precautions far simpler in their details than most of us imagine, and the whole is largely a matter of common sense, instead of dealing with the matter as an eccentric, if hygienic, fad. The country must, and can, indeed, easily, have clean milk if only the facts will sink into the public mind and a demand for it be created. The effort on the part of the "trade" will undoubtedly come if the consumer insists upon it.

Two inspectors from the Health Ministry are in daily attendance to give full technical advice to dairymen, farmers, and distributors. Other officials are at hand to help the general public, and a film illustrating the correct methods of production is also a part of this valuable demonstration.

